# Health-related quality of life and coping strategies adopted by COVID-19 survivors: A nationwide cross-sectional study in Bangladesh

**DOI:** 10.1371/journal.pone.0277694

**Published:** 2022-11-16

**Authors:** Mohammad Anwar Hossain, Rubayet Shafin, Md. Shahoriar Ahmed, Md. Shohag Rana, Lori Maria Walton, Veena Raigangar, Tasnim Ara, Md. Aminul Hoque Rasel, Mohammad Sohrab Hossain, Md. Feroz Kabir, Mir Raihanul Islam, Md. Nazmul Hasan, Md. Delowar Hossain, Farjana Sharmin Rumana, Iqbal Kabir Jahid

**Affiliations:** 1 Department of Microbiology, Jashore University of Science & Technology (JUST), Jashore, Bangladesh; 2 Department of Physiotherapy, Centre for the Rehabilitation of the Paralysed (CRP), Savar, Dhaka, Bangladesh; 3 Department of Physiotherapy, Bangladesh Health Professions Institute (BHPI), CRP-Savar, Dhaka, Bangladesh; 4 Handicap International- Humanity & Inclusion, Bangladesh Program, Ukhiya, Cox’s Bazar, Bangladesh; 5 Dhaka College of Physiotherapy, Dhaka, Bangladesh; 6 Department of Physical Therapy, School of Health Sciences, University of Scranton, Scranton, Pennsylvania, United States of America; 7 School of Sport and Health Sciences, University of Brighton, Brighton, United Kingdom; 8 Institute of Statistical Research and Training, University of Dhaka, Dhaka, Bangladesh; 9 Department of Physiotherapy & Rehabilitation, Jashore University of Science & Technology (JUST), Jashore, Bangladesh; 10 Poverty, Health and Nutrition Division, International Food Policy Research Institute, Washington, D.C., United States of America; 11 Department of Physiotherapy & Rehabilitation, Enam Medical College Hospital, Savar Union, Bangladesh; Yenepoya University Yenepoya Physiotherapy College, INDIA

## Abstract

**Introduction:**

This study aims to investigate the health-related quality of life and coping strategies among COVID-19 survivors in Bangladesh.

**Methods:**

This is a cross-sectional study of 2198 adult, COVID-19 survivors living in Bangladesh. Data were collected from previously diagnosed COVID-19 participants (confirmed by an RT-PCR test) via door-to-door interviews in the eight different divisions in Bangladesh. For data collection, Bengali-translated Brief COPE inventory and WHO Brief Quality of Life (WHO-QoLBREF) questionnaires were used. The data collection period was from October 2020 to March 2021.

**Results:**

Males 72.38% (1591) were more affected by COVID-19 than females 27.62% (607). Age showed significant correlations (p<0.005) with physical, psychological and social relationships, whereas gender showed only a significant correlation with physical health (p<0.001). Marital status, occupation, living area, and co-morbidities showed significant co-relation with all four domains of QoL (p<0.001). Education and affected family members showed significant correlation with physical and social relationship (p<0.001). However, smoking habit showed a significant correlation with both social relationship and environment (p<0.001). Age and marital status showed a significant correlation with avoidant coping strategies (p<0.001); whereas gender and co-morbidities showed a significant correlation with problem-focused coping strategies (p<0.001). Educational qualification, occupation and living area showed significant correlation with all three coping strategies(p<0.001).

**Conclusion:**

Survivors of COVID-19 showed mixed types of coping strategies; however, the predominant coping strategy was avoidant coping, followed by problem-focused coping, with emotion-focused coping reported as the least prevalent. Marital status, occupation, living area and co-morbidities showed a greater effect on QoL in all participants. This study represents the real scenario of nationwide health-associated quality of life and coping strategies during and beyond the Delta pandemic.

## Background

In Bangladesh, the COVID-19 pandemic has progressed rapidly overtime and the burden of the Delta variant entering from neighboring countries [1], in addition to lack of resources within Bangladesh, low vaccine availability, affordability, accessibility and implementation have added to the country’s devastating COVID-19 infection rates and death rates. As the country prepared for its fourth wave, the infection rate was estimated to be over9%% on September13, 2022 [2]. As of September12, 2022, in Bangladesh, the total samples tested were 14,794,855 of which 2014887 confirmed cases and 29,334 deaths [3]. The increased death in Bangladesh during this period was attributed due to the second wave, initially by South African Beta variant (B.1.351) [4] and Indian Delta variant (B.1.617.2 [5]. With 150 nations, since March 18, 2021, Bangladesh suspended all academic institutions [6] and from March 26, 2020, the Bangladeshi Government encouraged people to stay home to prevent the rapid spread of COVID-19. This long-time, infrequent lockdown that started from March 10, 2020 [7], coupled with the catastrophic impact of COVID-19, which may eventually cause acute respiratory syndrome, respiratory failure, heart failure, or even death, have a negative impact on people’s social and mental health and have a significant impact on increasing stress and anxiety for the general population. All these factors had a substantial negative impact on the Quality of life (QoL) [8–11].

Quality of life is a broad term and represents one’s overall physical, mental, social, and environmental satisfaction. Due to the loss of lives and livelihoods, COVID-19 has exacerbated psychosocial and socioeconomic insecurity among poor people by causing price hike of basic products, restriction of informal education, and the risk of a serious socio-economic and health crisis [12]. However, due to the shutdown of exports and imports, many people lost their jobs (for example, garment workers, corporate office employees, and foreign revenue declines) further affecting the quality of life (QoL) for people already struggling economically prior to the onset of COVID-19 [13]. Humans have shown great capacity for developing a variety of coping mechanisms for survival during and after catastrophic events. However, the extra burden of poverty on people during a catastrophic event has been shown to have cumulative negative impacts on the psychological coping strategies for people over long periods of time [14]. Coping methods are emotion-driven efforts to handle stress that has been linked to improved mental health and are necessary components to healing from trauma [15]. Studies have shown that the coping method adopted by individuals has a significant impact on how they experience anxiety and process behavioral responses [16]. Communication, avoidance and activities are some of the methods being used as Coping strategies. From the definition COPING is “Efforts to prevent or diminish threat, harm, and loss, or to reduce the distress that is often associated with those experiences” [17] which can be described as the broad terms "Approach-an issue is solved by controlling stress" and "Avoidant-a problem is solved by avoiding stress by reducing unpleasant emotions". Scholarly evidence shows that Approach Coping Strategy (APC) is more common in the Bangladeshi community than Avoidance Coping Strategy (AVC) [18]. To the best of our knowledge, there is a scarcity of empirical evidence concerning the effects of COVID-19 on coping and QoL among the patients recovering from this infectious disease. Therefore, in this study, we aimed to assess the comparison between the behavioral aspect of COPING strategies and the impact of QoL among Covid 19 populations in Bangladesh. The high-risk groups identified through this study could be targeted as the vulnerable groups who would require additional care and support from the government of Bangladesh during this crisis pandemic.

## Methodology

### Study design

This was a cross-sectional study of 2198 adult COVID-19 survivors collected from 14392 COVID-19 positive cases across all divisions of Bangladesh conducted on people who tested positive for Covid-19 from the time frame between October,2020 to March, 2021. All the participants tested positive or negative through RT-PCR nasopharyngeal swab under the national surveillance systems of COVID-19 located at the Directorate of General of Health Services (DGHS) in various laboratories throughout Bangladesh [19]. The RT-PCR test for SARS-CoV-2 has been documented as the gold standard and most of the countries, including Bangladesh, are using the RT-PCR for diagnosis the COVID-19 [20]. Inclusion criteria for this study were diagnosed COVID-19 with a minimum age of 18 years, the presence of persistent secondary problems following a positive diagnosis, and the presence of difficulty with usual activities of daily living (ADLs) [21]. Exclusion criteria included persistent fever, inability to participate due to illness, mental instability and refusal of permission.

### Sample size

**Fig 1:**
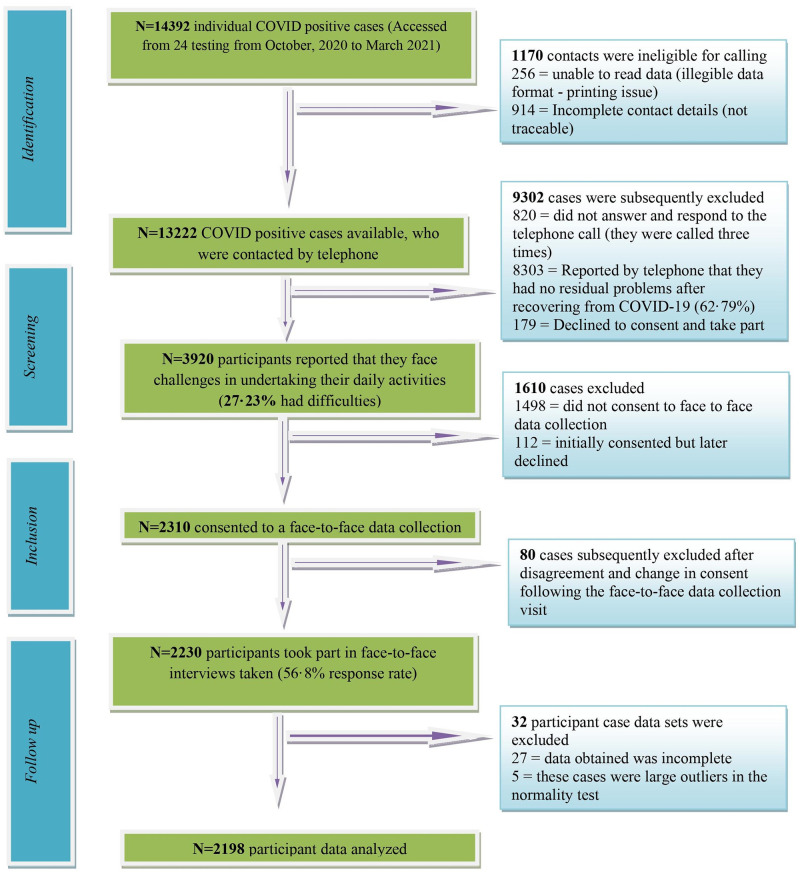
STROBE flow diagram of the study.

The sample size calculation was performed using “EPI INFO” software version 7.4.2.0 developed by the Center for Disease Control in the US. For the calculation. The reference figure of 20,14,887 was used (i.e., The total number of COVID-19 positive cases reported up to September 2022) [2] with a cluster figure of eight (the number of administrative divisions in Bangladesh) A calculation was then made with 50% of expected frequency, 5% margin of error, and 1.0 design effect. So, the desired sample size was generated with 99.99% confidence interval as a minimum of 1512 with 189 samples per cluster. Then a total of 2198 samples were recruited for analysis [Fig 1].

### Study procedure

A clear flow diagram of the study process had been produced in Fig 1 to meet the quality guidelines recommended by Strengthening the Reporting of Observational studies in Epidemiology (STROBE) [22]. Data were collected through appointed trained assessors from the Centre for the Rehabilitation of the Paralyzed (CRP). The research team initially reviewed the materials from World Health Organization (WHO), Directorate General of Health Services (DGHS), Ministry of Health and Family welfare, Bangladesh and made a framework of questionnaires. The questionnaire were initially drafted in English but later translated in Bangla by a researcher who had good knowledge in both languages and the validation process was followed as per WHO guidance [23]. Before data collection all assessors were comprehensively trained by the principal author regarding study protocols, precaution, adverse events, aims, ethical considerations, questionnaires and the possible outcomes. A pilot study was conducted with 20 participants, with face-to-face data collection and was undertaken at a convenient scheduled time for participants, after taking written consent from the participants. Informed consent and questionnaires were provided in paper format, with consent read aloud for every participant in their native language to assure full comprehension. During data collection, all the assessors adhered to the COVID-19 preventive precautions by utilizing personal protective equipment (PPE) and general health regulations set forth by the Bangladesh Government. Data was collected in paper format and then transferred into Excel Workbook for external data audit. After completion of the data audit, the data was analyzed in SPSS, version 20.0.

### Data collection and questionnaire

A phone call follow-up was conducted with all participants (N = 13,222) for any secondary complications after receiving a negative test result for COVID-19 [Fig 1]. A total 2198participants with secondary complications provided consent and completed the questionnaire. The first part of the questionnaire provided socio-demographic information and the comorbidity information, the second part provided Brief-COPE and WHOQoL-BREF. The Brief-COPE is a frequently used self-reported questionnaire that was developed to assess a broad range of coping strategies. It has 28 items questionnaires that describes the COPING responses in three domains (problem, emotion and avoidant focused). Each item in each domain is scored from possible options on an ordinal scale from one to four. The World Health Organization Quality of Life-BREF scale was used to determine QOL. The WHOQOL-BREF is a 26-item scale that is used to assess people’s quality of life. It is an abbreviated version of the WHOQOL-100 scale. It consists of four domains as well as a general health domain. Physical health (7 items), psychological health (6 items), social relationships (3 items), and the environment (8 domains). The final two items are from the general health domain, which enables respondents to score their overall satisfaction with their health and quality of life. The scale items are graded on a five-point Likert scale ranging from 1 (very dissatisfied/very poor) to 5 (very satisfied/very good), with higher scores indicating better quality of life [24]. Large values of KMO statistic (>0.8) for both WHO-QoL and Brief Cope questionnaire indicated that the sample was suitable for factor analysis. On the other hand, the reliability was determined by calculating Cronbach’s α coefficient. The coefficient was measured as 0.716 and 0.886 respectively, well above the minimum accepted threshold of 0.70 [25].

### Statistical testing

**Fig 2:**
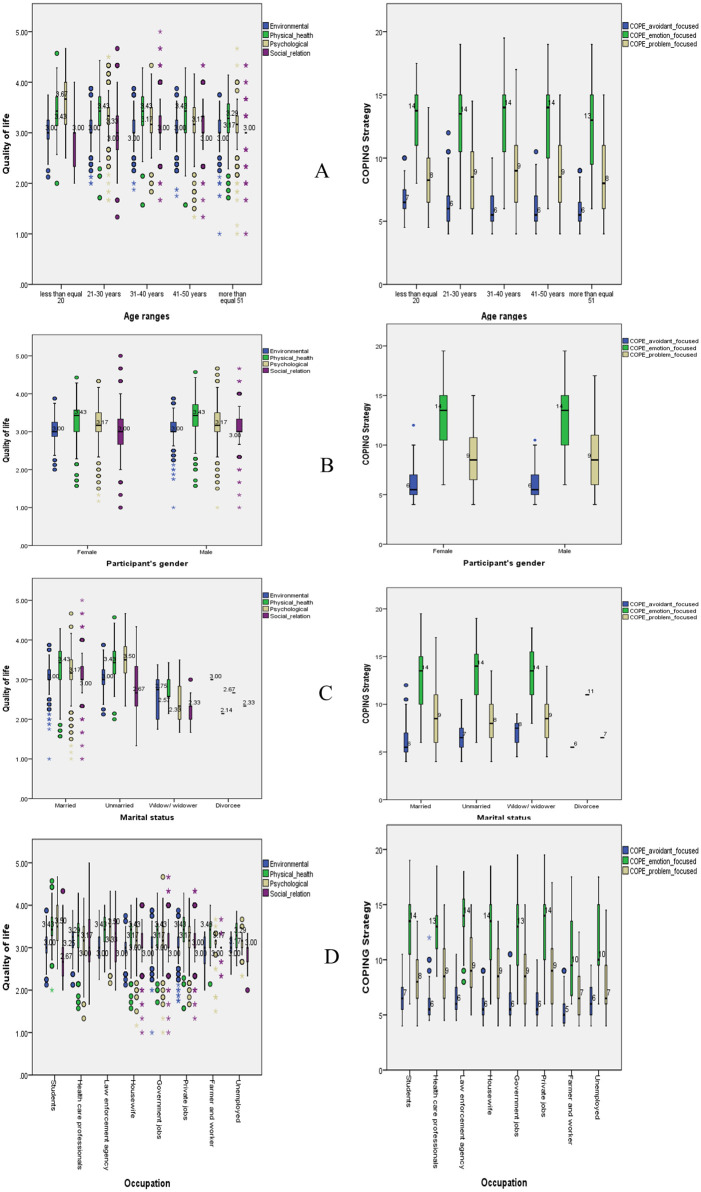
Box plot of COPING and QoL.

Data were analyzed using the Statistical Package for Social Science (SPSS) version 20.0 [26]. The Kaiser-Meyer-Olkin (KMO)analysis were done between WHO QoL and Coping, for data adequacy as well as normality for factor analysis. Descriptive analysis was performed for parametric socio-demographic, dependent variable and health and co-morbidities of the respondents (Table 1). In addition, multivariate analysis of variance (One-way MANOVA) statistics was performed for dependent variables between QoL and coping strategies (Tables 2 and 3). Population distribution is shown in the Box plot (Fig 2). The alpha value was set as p<0.05.

**Table 1:** Demographic, health situation and comorbidities characteristics of the analytic sample.

	Frequency (n)	Percentage (%)
**Age; Mean (38.01 ± 11.56)**		
≤20	72	3.3
21–30	607	27.6
31–40	714	32.5
41–50	469	21.3
51+	336	15.3
**Gender**		
Male	1,591	72.4
Female	607	27.6
**Division**		
Barisal	234	10.7
Chittagong	187	8.5
Dhaka	789	35.9
Khulna	177	8.1
Mymensingh	214	9.7
Rajshahi	184	8.4
Sylhet	212	9.7
Rangpur	201	9.1
**Residence**		
Rural	142	6.5
Semi-urban	1,510	68.7
Urban	546	24.8
**Educational Status**		
No or primary education	93	4.2
Secondary	265	12.1
Higher secondary	739	33.6
Bachelor or above	1,101	50.1
**Employment Status**		
Student	225	10.2
Health care worker	463	21.1
Teaching/ private/ Govt. job	346	15.7
Business	1,164	52.9
**Income**		
Less than 25000	344	15.7
25000–50000	1,322	60.2
More than 50000	532	24.2
**Marital status**		
Married	1890	86
Unmarried	308	14
**Family Size**		
Small	1,323	60.2
Large	875	39.8
Hospital admitted	329	14.9
Diagnosed COVID-19 in family	518	23.6
Smoking history	360	16.4
**Comorbidities**		
Heart disease	63	2.9
Hypertension	271	12.3
Lung disease	73	3.32
Diabetes Mellitus	240	10.9
Chronic Kidney disease	18	0.8
Liver disease	61	2.8
Anemia	59	2.7
Cancer	54	2.5
Depression	76	3.5
Osteoarthritis	41	1.9
Back pain	89	4.1
Rheumatoid arthritis	12	0.6

**Table 2:** Relationship in between demographic variables with WHO quality of life.

Variables	Physical health			Psychological			Social relationship			Environmental		
	Mean±SE	F	Partial η^2^	Mean±SE	F	Partial η^2^	Mean±SE	F	Partial η^2^	Mean±SE	F	Partial η^2^
**Age**												
less than equal 20	93.5±1.1	22.9***	.04	85.9±1.1	43.4***	.073	41.1±.5	26.6***	.046	94.8±.8	.9	.002
21–30 years	93.6±.4			80.2±.4			40±.2			95.5±.3		
31–40 years	94±.3			78.5±.3			39.5±.2			95.5±.2		
41–50 years	91.9±.4			76.2±.4			38.2±.2			95.1±.3		
more than equal 51	88.8±.5			73.8±.5			37.4±.2			94.9±.4		
**Gender**												
Female	90.6±.4	41.1***	.018	77.1±.4	7.1	.003	38.9±.2	1.6	.001	95±.3	1.5	.001
Male	93.4±.2			78.4±.2			39.2±.1			95.4±.2		
**Marital status**												
Married	92.4±.2	7.1**	.003	77.1±.2	131.1***	.056	38.8±.1	55.6***	.025	95.3±.2	.05	.000
Unmarried	93.9±.5			83.7±.5			40.9±.3			95.2±.4		
**Education**												
No formal Education	86.4±1.4	11.8***	.021	76.9±1.6	6.1***	.011	40.3±.8	14.7***	.026	90.3±1.1	19.2***	.034
Primary Education	93.3±1.2			76.8±1.2			37.5±.6			93.3±.9		
Secondary Education	91.7±.6			78.4±.6			38.8±.3			93.3±.4		
Higher secondary Education	91.5±.3			76.7±.4			38.3±.2			95.1±.2		
Bachelor or above	93.7±.3			78.9±.3			39.8±.1			96.2±.2		
**Occupation**												
Students	94.4±.6	13.1***	.040	85.4±.6	34.6***	.100	41.1±.3	18.3***	.056	95.7±.4	6.9***	.022
Health care professionals	87.5±.8			74.3±.8			40.4±.4			96.1±.6		
Law enforcement agency	93.1±.9			82.2±.9			41.6±.5			94.2±.7		
Housewife	90.1±.6			76.4±.6			38.6±.3			94.2±.4		
Government jobs	91.6±.5			75.8±.6			39.2±.3			94.9±.4		
Private jobs	93.6±.3			77.9±.3			38.7±.1			95.8±.2		
Farmer and worker	91.4±1.2			72.2±1.3			36.3±.7			90.3±.9		
Unemployed	91.2±1.5			72.9±1.5			36.5±.8			93.9±1.1		
**Living area**												
Rural	94.4±.8	8.8***	.008	79.9±.8	35.3***	.031	40.5±.4	114.2***	.094	90.5±.5	47.1***	.041
Semi Urban	92.9±.2			76.9±.2			38.2±.1			95.9±.2		
Urban	91.3±.4			80.7±.4			41.4±.2			94.9±.3		
**Family members affected**												
Affected family members	91.1±.4	19.4***	.009	78.2±.4	.2	.000	40.4±.2	48.5***	.022	94.5±.3	10.0	.005
Unaffected family members	93.1±.2			77.9±.2			38.7±.1			95.6±.2		
**Smoking history**												
Smokers	92.5±.5	.1	.000	78.2±.5	.1	.000	40.2±.2	22.8***	.010	93.1±.3	48.6***	.022
Non-smoker	92.6±.2			77.9±.2			38.9±.1			95.7±.2		
**Comorbidities**												
No comorbidity	93.9±.2	60.4***	.076	79.1±.2	43.7***	.056	39.2±.1	6.7***	.009	95.6±.2	11.2***	.015
one comorbidity	90.8±.5			77.4±.6			39.8±.3			95.6±.4		
two comorbidities	86.9±.7			72.1±.7			37.9±.4			94.3±.5		
more than two comorbidities	86.7±.8			72.8±.8			38.4±.4			92.3±.6		

Significant relationship values with a minimum of 5% margin of error are bolded and marked as

* p<·05,

** p<·01,

*** p<·001

**Table 3:** Relationship in between demographic variables with COPING strategies.

Variables	Problem focused			Emotion focused			Avoidant focused		
	Mean±SE	F	Partial η^2^	Mean±SE	F	Partial η^2^	Mean±SE	F	Partial η^2^
**Age**									
less than equal 20	7.5±.7	1.2	.002	11.9±.8	2.3	.004	6.7±.4	3.3**	.006
21–30 years	7.2±.6			11.9±.7			6.5±.3		
31–40 years	7.4±.6			12.3±.7			6.6±.3		
41–50 years	7.3±.6			12.3±.7			6.6±.3		
more than equal 51	7.2±.6			11.9±.7			6.3±.3		
**Gender**									
Female	7.1±.6	7.7**	.004	12.2±.7	1.2	.001	6.6±.3	3.1	.001
Male	7.5±.6			11.9±.7			6.4±.3		
**Marital status**									
Married	8.6±.1	3.1	.001	12.6±.1	6.1	.003	5.9±.1	45.6***	.020
Unmarried	8.3±.2			13.1±.2			6.5±.1		
**Education**									
No formal Education	6.6±.7	7.1***	.013	11.7±.8	5.8***	.011	6.6±.4	5.1***	.009
Primary Education	6.7±.7			11.1±.8			6.1±.3		
Secondary Education	7.4±.6			12.2±.7			6.7±.3		
Higher secondary Education	7.9±.6			12.8±.7			6.8±.3		
Bachelor or above	7.9±.5			12.5±.7			6.5±.3		
**Occupation**									
Students	7.3±.6	2.2*	.007	12.2±.7	3.3**	.011	6.7±.3	3.8***	.012
Health care professionals	6.9±.6			11.7±.7			6.4±.3		
Law enforcement agency	7.6±.6			13.1±.7			6.8±.3		
Housewife	7.6±.6			11.6±.7			6.2±.3		
Government jobs	7.1±.6			12±.7			6.5±.3		
Private jobs	7.5±.6			12.3±.7			6.4±.3		
Farmer and worker	7.5±.7			11.4±.8			6±.3		
Unemployed	7.1±.7			11.9±.8			6.9±.3		
**Living area**									
Rural	7±.6	18.1***	.016	11.8±.7	51.2***	.045	6.4±.3	46.7***(.000)	.041
Semi Urban	7±.5			11.3±.7			6.2±.3		
Urban	7.8±.5			13.1±.7			6.9±.3		
**Family members affected**									
Affected family members	7.1±.5	4.7*	.002	12.3±.7	10.8***	.005	6.5±.3	2.7	.001
Unaffected family members	7.4±.5			11.8±.7			6.4±.3		
**Smoking history**									
Smokers	6.8±.5	40.4***	.018	12±.7	.5	.000	6.6±.3	15.9***	.007
Non smoker	7.7±.5			12.1±.7			6.3±.3		
**Comorbidities**									
No comorbidity	8.6±.1	7.1***	.010	12.7±.1	1.7	.002	6.1±.1	1.6	.002
one comorbidity	8.4±.1			12.4±.1			6.1±.1		
two comorbidities	8.1±.2			12.3±.2			5.8±.1		
more than two comorbidities	7.8±.2			12.3±.2			5.9±.1		

Significant relationship values with a minimum of 5% margin of error are bolded and marked as

* p<·05,

** p<·01,

*** p<·001

The differences in the usage of the three coping strategies were tested using one-way Multivariate analysis of variance (MANOVA) procedure.

## Results

### Demographic characteristics of the analytic sample

The socio-demographic and comorbidities characteristics of the participants are demonstrated in Table 1. A total 2198 participants aged 18 years to 86 years of age responded to the survey. Most of the respondents 32.5% (n = 714) were from the age group (31–40 years). Male respondents were 72.4% (n = 1591) and female 27.6% (n = 607). Regional disaggregation of the samples showed that most of the respondents were 35.9% (n = 789) from the Dhaka division and over two third of them were 68.7% (n = 1510) living in the semi-urban areas. More than half of the participants were (52.9%, n = 1164) involved in business activities, and 50.1% (n = 1101) reported completion of a bachelor’s degree. Around 15.7% (n = 1322) of the respondents reported income less than 25000TK per month and the majority (84.9%, n = 1868) of the respondents reported being married. Almost 60.2% (n = 1323) of the sample belong to a small family size. A small number of the participants, around 16.4% (n = 360) reported were previous smoking status (Table 1).

### Comorbidities of the respondents

Approximately15% (n = 329) of the respondents were admitted into hospitals and most of them, 85% (n = 1869) were not hospitalized. Almost one-fourth of the respondents have at least one family member who was diagnosed with COVID-19 23.6% (n = 518). Comorbidities represented included: hypertension was found to have the highest prevalence at 12.33% (n = 271) followed by Diabetes Mellitus reported as second highest at 10.9% (n = 240). All other comorbidities reported almost the same prevalence rate (Table 1).

### Relation of demographic with quality of life

There was a significant correlation between sociodemographic variables and quality of life of COVID-19 survivors (Table 2). Age showed significant correlation with physical health (F:22.9, partial ƞ^2^: .04 and p < .001), psychological health (F:43.4, p < .001), and social relationship (F:26.6, p < .001), where respondents at 31–40 years old had good physical health (Mean±SE: 94±0.3) than other age groups, but respondents at less than 20 years older had good psychological (Mean±SE: 85±0.9) and social relationship (Mean±SE: 41.1±0.5) than other age groups. Gender showed significant correlation with only physical health (F:41.1, p < .001), where male respondents had good physical health (Mean±SE: 93.4±0.2) than female respondents. Marital status had found significant correlation with physical health (F:7.1, p < .01), psychological health ((Mean±SE: 93.4±0.2)) and social relationship (F:55.6, p < .001); where unmarried respondents had good physical health (Mean±SE: 93.9±0.5), psychological health (Mean±SE: 83.7±0.5), and social relationship (Mean±SE: 40.9±0.3) than married respondents. Educational qualification had found significant correlation with all four quality of domains (Physical health:F:11.8, p < .001; psychological health: F:6.1, p < .001; social relationship: F:14.7, p < .001;environment: F 19.2, p < .01); where higher educated respondents who completed bachelor or above degree had good physical health (Mean±SE: 93.7±0.3), psychological health (Mean±SE: 78.9±0.3), and environmental (Mean±SE: 96.2±0.2) but no formal education group respondents hadgood in social relationship (Mean±SE: 40.3±0.8) than other educational groups of the respondents. Occupational characteristics had found significant with physical health (F:13.1, p < .001), psychological health (F:34.6, p < .001), social relationship (F:18.3, p < .001) and environmental (F:6.9, p < .001), where students had good in physical health (Mean±SE:94.4±0.6) and psychological health (Mean±SE:85.4±0.6) but law enforcements had good in social relationship (Mean±SE:41.6±0.5) and health professionals had higher score in environmental QoL (Mean±SE: 96.1±0.6). Living area found significant correlation with all four domains (Physical health F:8.8, p < .001; psychological health F: 35.3, p < .001; social relationship F: 114.2, p < .01; environmental F: 47.1, p < .01) of quality of life. Rural respondents had more score on physical health (Mean±SE: 91.3±0.4) than others; where urban respondents had higher score on psychological (Mean±SE: 80.7±0.4) and social relationship (Mean±SE: 41.4±0.2) than other living area groups while semi-urban respondents had more score on environmental (Mean±SE: 95.9±0.2) quality of life than others. Family member affected had found significant correlation with physical health (F: 19.4, p < .001) and social relationship (F: 48.5, p < .001) domains of quality of life. Where unaffected family members group had better physical health (Mean±SE: 93.1±0.2) quality of life than affected family members group while affected family members group had better score on social relationship (Mean±SE: 40.4±0.2) domain of quality of life. Smoking history had found significance with social relationship (F: 22.8, p < .001) and environmental domain of quality of life (F: 95.7, p < .001) while smokers’ group had higher score on social relationship (Mean±SE: 40.2±0.2) than non-smokers group and non-smokers group had more score on environmental (Mean±SE: 95.7±0.2) domain of quality of life than smokers’ group. Having comorbidities had found significance with physical health (F: 60.4, p < .001), psychological health (F: 43.7, p < .001), social relationship (F: 6.7, p < .001) and environmental (F: 11.2, p < .01) domains of quality of life. Where having no comorbidities group had higher score on physical health (Mean±SE: 93.9±0.2) and psychological health (Mean±SE:79.1±0.2) than others having comorbidities groups; while having one comorbidity group had, higher score on social relationship (Mean±SE: 39.8±0.3) and no comorbidity and having one comorbidity had similarly higher score (Mean±SE: 95.6±0.4) on environmental health domains of quality of life. (Table 2).

### Relation of demographic with COPING strategies

From Table 3 it was apparent that sociodemographic variables had significant correlation with COPING strategies. Respondents age had found a significant correlation with Avoidant coping strategy (F: 3.3, p < .01). From the age category, respondents who was less than 20 years old or younger respondents had higher mean score (6.69±3.66) to the avoidant coping than the other age groups. Gender had significant correlation with problem focused coping strategy (F: 7.7, p < .01) where male participants had higher mean scores than female (7.5±0.6) to problem focused coping. Marital status had significant relation with avoidant focused coping (F: 45.6, p < .001) strategy and among the marital status category unmarried participants had highest mean score to the avoidant coping (6.52±0.1) strategies. Educational qualification had significant correlation with all three-problem focused (F:7.1, p < .001), emotion focused (F:5.8, p < .001) and avoidant focused (F:5.1, p < .001) coping strategies. From the respondents, who were bachelor or above had highest mean score to the problem focused coping (7.9±0.59) whereas respondents who completed higher secondary had highest mean score to the emotion focused coping and avoidant coping (12.8±0.7; 6.8±0.3) strategies. Occupational status had also significant correlation with all three-problem focused (F:2.2, p < .05), emotion focused (F:3.3, p < .01) and avoidant focused (F:3.8, p < .001) coping strategies. From the occupational categories housewives had highest mean score (Mean±SE:7.6±0.6) to problem focused coping strategy and respondents who were from law-enforcement agency had highest mean score (Mean±SE:13.1±0.7) to the emotion focused coping and those who were unemployed had highest mean score to the avoidant coping strategy (Mean±SE:6.9±0.3). Living area had found significant correlation with problem focused (F:18.1, p < .001), emotion focused (F:51.2, p < .001) and avoidant focused (F:46.7, p < .001) coping strategies, where urban respondents had higher score in all three coping strategies and their Mean±SE were 7.8±0.5, 13.1±0.7 and 6.9±0.3 respectively. Affected family members had also found significance with problem focused (F:4.7, p < .05) and emotion focused (F:10.8, p < .001) coping strategies where unaffected family members group had showed more problem focused coping strategies (Mean±SE: 7.4±0.5) than affected family members group but affected family members group had shown more emotion focused coping (Mean±SE: 12.3±0.7) than unaffected family members group. Smoking history had found significance with problem focused (F:40.4, p < .001) coping and avoidant focused coping (F:15.9, p < .001) strategies where non-smokers, had higher score on problem focused coping (Mean±SE: 7.7±0.5) but smokers had higher score on avoidant focused coping strategies (Mean±SE: 6.6±0.3). Having comorbidities had found significant correlation with problem focused coping (F:7.1, p < .001) while having no comorbidity showed highest score on problem focused (Mean±SE: 8.6±0.1) coping than other groups (Table 3).

### Correlation between COPING strategies and Quality of Life (QoL)

Table 4 showed some weak to moderate correlations that were found between COPING and the QoL domains. Problem focused coping was associated with psychological (r = .165, p < .001 and social relation (r = 0.061, p < .01). Emotion focused coping correlated with psychological (r = .104, p <0.001) and social relation (r = .150, p < 0.001) and negatively associated with physical health (r = -.090, p <0.001) and environment (r = −.236, p < 0.001). Avoidant focused coping was negatively associated with physical health (r = -.220, p <0.001) and environment construct (r = −.217, p > 0.001) (Table 4).

**Table 4:** Correlation between COPING and QoL by Pearson correlation test.

	Problem focused	Emotion focused	Avoidant focused	Physical health	Psychological	Social relation	Environment
Problem focused	1	.678***	.330***	.017	.165***	.061**	-.027
Emotion focused		1	.572***	-.090***	.104***	.150***	-.236***
Avoidant focused			1	-.220***	.056**	.039	-.217***
Physical health				1	.559***	.239***	.315***
Psychological					1	.243***	.289***
Social relation						1	.058
Environment							1

Significant relationship values with a minimum of 5% margin of error are bolded and marked as

* p<·05,

** p<·01,

*** p<·001

### Age, gender, marital status and occupational wise distribution of WHO-QoL and COPING strategies

From the boxplot Fig 2(A) In quality-of-life domains, participants less than 20 years old had higher score on psychological health (Mean: 3.67), On the other hand, in COPING strategies, participants in all the age groups had similar higher score on emotion focused COPING strategies. Fig 2(B) described the gender-based quality of life among the participants where female and male had higher similar score on physical health (Mean: 3.43) and in COPING strategies male (Mean:14) and female (Mean:14) both had the similar high mean score on emotion focused COPING strategies. Fig 2(C) married participants had higher mean score (Mean:3.43) on physical health, unmarried respondents had higher mean score (Mean:3.50) on psychological health. COPING strategies participants who were married and unmarried had similar high mean score (Mean: 14). Fig 2(D) Unemployed had the highest mean score on physical health (Mean: 4.39); On coping strategies, students, health care professionals, law enforcement agency, housewife, government employer, private jobs, farmers and unemployed all had quite similar mean score on emotion focused coping.

## Discussion

The demographic statistics [Table 1] showed most of the participants were in their third and fourth decade of life 32.48% (n = 714), and that majority of the participants were male 72.4% (n = 1591). A study from China showed similar findings, where males were more affected by COVID-19 than females [27]. The prevalence 68.70% of COVID-19 was found to be higher in the semi-urban areas, meaning their residential area was in the district or sub district of Upazilla Level. Education data showed that out of 2198 participants, half of the sample 50.09% (n = 1101) completed their bachelor’s degree at minimum, and participants involved in business as an occupation included a large proportion at52.96% (1164). Similar demographic characteristics were evident from another article where most of the participants were in their third and fourth decade of life, males, completed bachelor’s degree and residing in an urban area [28]. From the total data, 48.11 percent had co-morbidities, with hypertension accounting for the highest 12.33 percent and diabetes mellitus accounting for the second highest 10.92 percent. Symptom-responses were more prevalent, in our study, among those with higher education and mainly businessmen.

Quality of Life (QoL) is a well-known term used by health care experts all over the world to assess any disease outcome. COVID-19 has a significant impact on people’s health-related QoL [29]. A significant correlation was found between sociodemographic and QoL of COVID-19 survivors. Age showed significant correlation with physical, psychological, and social relationships, but was not significantly correlated with the environment. The highest mean score showed people living in their third decade of life had better physical health outcomes while the age group 20 and below had better psychological and social relationship status. Gender showed men reported slightly better QoL in all four domains than women. This was similar to a previous Jordanian study where women reported higher rates of depression and lower QoL compared to men [29]. Marital status reported that unmarried participants have better psychological and social relationships than married participants. Similar findings were found in a previous study which stated that marriage initiated a process of increasing reliance on, and time spent with the partner and family relatives. Furthermore, it resulted in less reliance on and time with friends and non-relatives’ peers so, married people tended to participate in fewer and more family-focused activities rather than social activities [30]. Bachelor or above degree holders showed better physical, psychological, and environmental quality of life, whereas people with no formal education represented better social relationship score. Previous studies showed higher levels of knowledge and education were all linked to positive attitudes and health preventive practices during COVID-19 [31]. Occupation mean score showed students had significant physical, psychological, social and environmental score which means in Bangladesh student poses overall good quality of life. Though evidence from developed countries shows students have negative impact with quality of life during post COVID-19 situation [32]. Psychological and social relationship scores showed significant relationships with people who live in urban areas. In terms of administrative divisions, people living in Chittagong reported higher psychological, social, and environmental mean scores than all other divisions. Respondents with no reported comorbidities also showed better physical, psychological and environmental scores compared with respondents with at least one or more comorbidities.

Furthermore, this community-based study showed coping strategies and QoL for COVID-19 survivors’ of 2198 participants derived from both rural and urban area from the eight divisions of Bangladesh. It represents, age ≥ 20 years old, and shows a significant correlation with avoidant coping strategies which indicates their physical or cognitive efforts to disengage from the stressor. Additionally, a low mean score with the age of 41–50 years is indicative of adaptive coping. Problem focused coping showed a significant correlation with male gender. However, female was more prone to emotion focused coping in this study. This possible reason is related to the accepted cultural expectation in Bangladesh that female gender identity is connected to intuitive and emotional style coping mechanisms and cumulative burden for everyday stressors [33] Previous evidence showed that during the SARS outbreak, more women than men sought counseling for emotional reasons [34]. Also, marital status had a significant and strong correlation with avoidant coping with a means core higher for widow or widower compared to all other categories. In the current study, education, occupation, living area and administrative division found significant co-relations with problem, emotion and avoidant coping strategies. Furthermore, higher mean score on problem focused associated with higher education levels, housewife, urban area and Chittagong division. According to previous research, higher education is associated with more positive coping methods, regardless of gender. However, women, either married, single or separated/divorced, overall, reported a higher burden of work during COVID-19, resulting in more negative coping strategies and poorer health outcomes [35]. Family members shows significant co-relation with problem and emotion focused coping, whereas unaffected family members reported high mean scores related to problem solving approach. But, affected family members represented regulation of emotions that were associated with specific stressful situations. Previous studies showed that family was negatively associated with coping strategies [17].

Correlation between COPING and QoL showed problem focused coping strategies were positively correlated with psychological and social relations which indicate they are able to manage more stressful situations. Emotion focused showed positive correlation with psychological and social realms but were negatively associated with physical health and environmental health, indicating they are capable of regulating emotions associated with the stressful situation. Avoidant focused coping strategy was positively correlated with psychological health indicating better stressor coping skills; however, it was also negatively associated with physical and environment, indicative of a more adaptive coping strategy. Another study showed individuals who employed an avoidance coping technique had lower levels of wellbeing and QOL, which is often considered a maladaptive coping strategy [31]. From the box plot it shows the second decade of life had a higher mean score with physical health, and all age groups had a higher mean score with emotion focused. Both male and female QoL scores were identical in terms of physical health, but coping strategy represents better score in terms of emotional health. Previous evidence showed women poses more negative impact on the psychological health compare with men [32]. Married individuals had a higher mean score for physical health and unmarried participants had a higher mean score for psychological health, but all participants had a high mean score for avoidant coping. Another study showed individuals that were unmarried or divorced also reported a lower quality of life, possibly related to cognitive stressors [36]. Patients have little adapt with the rapid progression of COVID-19. Outcome shows female demonstrates emotion-focused coping while male shows problem-focused coping; in addition, male has greater QoL in all four dimensions than female. In these conditions, women health in Bangladesh most of the time neglected, therefore interdisciplinary team could help with diagnoses, therapy, and prevention. Attention should be made to women’s mental health policies, considering the "Rehabilitation" in care and not to delay diagnosis and treatment.

## Limitation

We evaluated HRQoL and coping strategies using self-report questionnaires. Self-reports may lead to an overestimation of psychological suffering, although they are frequently used and widely validated. However professional assessment of psychological disorders requires consultation with a psychologist or psychiatrist. In addition, coping strategies and HRQoL were comparable between COVID-19 induced and the sample of non-COVID 19 responders to see the impact on the overall quality of life and psychological distress. Further research could be done to find the causative reason behind the higher prevalence rate.

## Conclusion

According to our study, during the COVID-19 pandemic fourth wave, where long periods of quarantine and lockdown were enforced across Bangladesh, there was a high report of anxiety and poor coping strategies which was directly related to psychological, emotional, physical and cognitive health outcomes and decreased quality of life for respondents across the eight Districts in Bangladesh. In addition, our study identified a higher prevalence of COVID-19 in semi-urban areas. Education, occupation and living area showed problem focused coping strategy. Education plays an important role and higher education is associated to more positive coping methods. Men had a higher quality of life and were more problem-oriented, whereas women were more emotion-focused on their coping. The government should place a greater emphasis on education and women’s health and advocate on health promotion strategies for the vulnerable female population.

## Supporting information

S1 File Questionnaire for the study (English version) translated and validated in Bangla. (DOCX)

## References

[1] Hasan M, Islam M, Alam A, Sarkar S, Rahman M, Islam O et al. Initial reports of the SARS-CoV -2 Delta variant (B.1.617.2 lineage) in Bangladeshi patients: Risks of cross-border transmission from India. Health Science Reports. 2021;4(3). doi: 10.1002/hsr2.366 34522791 PMC8425784

[2] COVID-19 [Internet]. Dashboard.dghs.gov.bd. 2021 [cited 26 July 2021]. http://dashboard.dghs.gov.bd/webportal/pages/covid19.php

[3] COVID-19 [Internet]. Dashboard.dghs.gov.bd. 2021 [cited 15 November 2021]. http://dashboard.dghs.gov.bd/webportal/pages/covid19.php

[4] Rahman M, Shirin T, Rahman S, Rahman M, Hossain M, Khan M et al. The emergence of SARS-CoV-2 variants in Dhaka city, Bangladesh. Transboundary and Emerging Diseases. 2021 10.1111/tbed.14203 PMC8447378 34170629

[5] Hasan M, Islam M, Alam A, Sarkar S, Rahman M, Islam O et al. Initial reports of the SARS-CoV -2 Delta variant (B.1.617.2 lineage) in Bangladeshi patients: Risks of cross-border transmission from India. Health Science Reports. 2021;4(3). doi: 10.1002/hsr2.366 34522791 PMC8425784

[6] Khan A, Sultana M, Hossain S, Hasan M, Ahmed H, Sikder M. The impact of COVID-19 pandemic on mental health & wellbeing among home-quarantined Bangladeshi students: A cross-sectional pilot study. Journal of Affective Disorders. 2020;277:121–128. doi: 10.1016/j.jad.2020.07.135 32818775 PMC7410816

[7] Anwar S, Nasrullah M, Hosen M. COVID-19 and Bangladesh: Challenges and How to Address Them. Frontiers in Public Health. 2020;8. doi: 10.3389/fpubh.2020.00154 32426318 PMC7203732

[8] Sultana S, Shafique I, Majeed N, Jamshed S, Shahani A, Qureshi F. Impact of Covid-19 outbreak on psychological health–The case of Bangladesh. Heliyon. 2021;7(4):e06772. doi: 10.1016/j.heliyon.2021.e06772 33948510 PMC8080049

[9] Serafini G, Parmigiani B, Amerio A, Aguglia A, Sher L, Amore M. The psychological impact of COVID-19 on the mental health in the general population. QJM: An International Journal of Medicine. 2020;113(8):531–537. doi: 10.1093/qjmed/hcaa201 32569360 PMC7337855

[10] Brooks S, Webster R, Smith L, Woodland L, Wessely S, Greenberg N et al. The Psychological Impact of Quarantine and How to Reduce It: Rapid Review of the Evidence. SSRN Electronic Journal. 2020. doi: 10.2139/ssrn.3532534 PMC7158942 32112714

[11] Salari N, Hosseinian-Far A, Jalali R, Vaisi-Raygani A, Rasoulpoor S, Mohammadi M et al. Prevalence of stress, anxiety, depression among the general population during the COVID-19 pandemic: a systematic review and meta-analysis. Globalization and Health. 2020;16(1). doi: 10.1186/s12992-020-00589-w 32631403 PMC7338126

[12] Bodrud-Doza M, Shammi M, Bahlman L, Islam A, Rahman M. Psychosocial and Socio-Economic Crisis in Bangladesh Due to COVID-19 Pandemic: A Perception-Based Assessment. Frontiers in Public Health. 2020;8. doi: 10.3389/fpubh.2020.00341 32676492 PMC7333562

[13] Truelove S, Abrahim O, Altare C, Lauer S, Grantz K, Azman A et al. The potential impact of COVID-19 in refugee camps in Bangladesh and beyond: A modeling study. PLOS Medicine. 2020;17(6):e1003144. doi: 10.1371/journal.pmed.1003144 32544156 PMC7297408

[14] Gellman MD, Turner JR, editors. Encyclopedia of behavioral medicine. New York, NY, USA: Springer; 2013.

[15] Lardone A, Sorrentino P, Giancamilli F, Palombi T, Simper T, Mandolesi L et al. Psychosocial variables and quality of life during the COVID-19 lockdown: a correlational study on a convenience sample of young Italians. PeerJ. 2020;8:e10611. doi: 10.7717/peerj.10611 33384910 PMC7751426

[16] Adasi G, Amponsah K, Mohammed S, Yeboah R, Mintah P. Gender Differences in Stressors and Coping Strategies Among Teacher Education Students at University of Ghana. Journal of Education and Learning. 2020;9(2):123.

[17] Encyclopedia of Behavioral Medicine. 2013;.

[18] Hossain K, Saunders K, Sakel M, Walton L, Raigangar V, Uddin Z et al. Coping with COVID-19 Pandemic: A Population-Based Study in Bangladesh. 2021.

[19] [Internet]. Dghs.gov.bd. 2021 [cited 15 November 2021]. https://dghs.gov.bd/images/docs/Notice/rt_pcr_lab.pdf

[20] PCR Test for COVID-19: What It Is, How Its Done, What The Results Mean [Internet]. Cleveland Clinic. 2021 [cited 15 November 2021]. https://my.clevelandclinic.org/health/diagnostics/21462-covid-19-and-pcr-testing.

[21] Edemekong PF, Bomgaars DL, Sukumaran S, Levy SB. Activities of daily living. InStatPearls [Internet] 2021 Sep 26. StatPearls Publishing. 29261878

[22] Vandenbroucke J, von Elm E, Altman D, Gøtzsche P, Mulrow C, Pocock S et al. Strengthening the Reporting of Observational Studies in Epidemiology (STROBE): Explanation and Elaboration. PLoS Medicine. 2007;4(10):e297. doi: 10.1371/journal.pmed.0040297 17941715 PMC2020496

[23] Younan L, Clinton M, Fares S, Samaha H. The translation and cultural adaptation validity of the Actual Scope of Practice Questionnaire. Eastern Mediterranean Health Journal. 2019;25(3):181–188. doi: 10.26719/emhj.18.028 31054228

[24] Vahedi S. World Health Organization Quality-of-Life Scale (WHOQOL-BREF): analyses of their item response theory properties based on the graded responses model. Iranian journal of psychiatry. 2010;5(4):140. 22952508 PMC3395923

[25] IBM Corp. Released 2011. IBM SPSS Statistics for Windows, Version 20.0. Armonk, NY: IBM Corp.

[26] García F, Barraza-Peña C, Wlodarczyk A, Alvear-Carrasco M, Reyes-Reyes A. Psychometric properties of the Brief-COPE for the evaluation of coping strategies in the Chilean population. Psicologia: Reflexão e Crítica. 2018;31(1). doi: 10.1186/s41155-018-0102-3 32026069 PMC6967273

[27] Islam M, Riaz B, Islam A, Khanam F, Akhter J, Choudhury R et al. Risk factors associated with morbidity and mortality outcomes of COVID-19 patients on the 28th day of the disease course: a retrospective cohort study in Bangladesh. Epidemiology and Infection. 2020;148. doi: 10.1017/S0950268820002630 33115547 PMC7653486

[28] Juran L, Trivedi J. Women, Gender Norms, and Natural Disasters in Bangladesh. Geographical Review. 2015;105(4):601–611. doi: 10.1111/j.1931-0846.2015.12089.x

[29] Sun F, Kosberg J, Kaufman A, Leeper J. Coping Strategies and Caregiving Outcomes Among Rural Dementia Caregivers. Journal of Gerontological Social Work. 2010;53(6):547–567. doi: 10.1080/01634372.2010.496823 20658420

[30] Shapiro A, Keyes C. Marital Status and Social Well-Being: Are the Married Always Better Off?. Social Indicators Research. 2007;88(2):329–346.

[31] Ausín B, González-Sanguino C, Castellanos M, Muñoz M. Gender-related differences in the psychological impact of confinement as a consequence of COVID-19 in Spain. Journal of Gender Studies. 2020;30(1):29–38. doi: 10.1080/09589236.2020.1799768

[32] Gao J, Wang F, Guo S, Hu F. Mental Health of Nursing Students amid Coronavirus Disease 2019 Pandemic. Frontiers in Psychology. 2021;12. doi: 10.3389/fpsyg.2021.699558 34475837 PMC8407077

[33] Gao WB, Chen ZY, Wang YN. Analysis on the influence and change trend of public mentality during Sars epidemic. Chinese Mental Health Journal. 2003;17(9):594–6.

[34] Berry E, Bachar E, Baras M, De Geest S. Correlates of coping based on the concept of the sociotype: a secondary data analysis of an Israeli National Survey. Health Psychology and Behavioral Medicine. 2017;5(1):177–196. doi: 10.1080/21642850.2017.1286497

[35] Hossain M, Jahid M, Hossain K, Walton L, Uddin Z, Haque M et al. Knowledge, attitudes, and fear of COVID-19 during the Rapid Rise Period in Bangladesh. PLOS ONE. 2020;15(9): e0239646. doi: 10.1371/journal.pone.0239646 32970769 PMC7514023

[36] Li L, Wang S. Prevalence and predictors of general psychiatric disorders and loneliness during COVID-19 in the United Kingdom. Psychiatry Research. 2020;291:113267. doi: 10.1016/j.psychres.2020.113267 32623266 PMC7326403

[37] WMA—The World Medical Association-Declaration of Helsinki [Internet]. Wma.net. 2021 [cited 16 November 2021]. https://www.wma.net/what-we-do/medical-ethics/declaration-of-helsinki/

