# Supplementary material for: Health-related quality of life and coping strategies adopted by COVID-19 survivors: A nationwide cross-sectional study in Bangladesh
Source: PLoS One. 2022 Nov 16;17(11):e0277694. doi: 10.1371/journal.pone.0277694 (PMC9668139; doi:10.1371/journal.pone.0277694)
Supplement: S1 File — (DOCX) [file pone.0277694.s001.docx]

**Questionnaire for the study (English version) translated and validated in Bangla**

**Personal details**

| Identification number: |
| --- |
| Initial of respondent: |
| Mobile number: Own- family member: |
| Permanent address |
| Present address |
| Code Number |
| Do you consent to participate in the study: Yes/No |
| Interviewer Signature with date: |

**1. Socio-demographic information:**

| **Question and filters** | **Response** |  |
| --- | --- | --- |
| 1.1 | Age | ………….. years |
| 1.2 | Gender  (Put √ on your answer) | 1= Male 2=Female |
| 1.3 | Marital status  (Put √ on your answer) | 1=Married  2=Unmarried 3=Widow/widower 4= Divorcee |
| 1.4 | Educational status  (Put √ on your answer) | 1= No formal education  2=Primary education 3=Secondary education 4=Higher secondary 5=Bachelor or above |
| 1.5 | Occupation  (Please write) |  |
| 1.8 | Living area  (Put √ on your answer) | 1=Rural  2=Semi-urban 3=Urban |
| 1.9 | Average monthly family income  (Please write) |  |
| 1.10 | Number of family members  (Please write) |  |

**2. Covid-19 related information:**

Before starting please mention your Blood group:

| 2.1 | When did you diagnose COVID positive?  (Please write the date) |  |
| --- | --- | --- |
| 2.2 | How long you were in isolation?  (Please write) | ………….. days |
| 2.3 | Had you been admitted tothe hospital?  (Put √ and write your answer) | 1= Yes  2= No  (if yes, mention the duration…………… days) |
| 2.4 | Days from symptom onset to hospital admission  (Please write) | ……………. days |
| 2.5 | Diagnosed COVID 19 in the family?  (Put √ and write your answer) | 1= Yes  2= No  (if yes, mention the number of your affected family members…………….) |
| 2.6 | Diagnosed COVID 19 inthe community?  (Put √ on your answer) | 1= Yes  2= No |
| 2.7 | Diagnosed COVID 19 in the working area?  (Put √ on your answer) | 1= Yes  2= No |
| 2.8 | Did you have a smoking history before diagnosed COVID positive? (Put √ on your answer) | 1= Yes  2= No |
| 2.9 | Did you have travel history from abroad before diagnosed COVID positive?  (Put √ and write your answer) | 1= Yes  2= No  (if yes, mention the name of the country………………….. |
| 2.10 | Did you visit the lockdown area/ affected area in Bangladesh before diagnosed COVID positive?  (Put √ and write your answer) | 1= Yes  2= No  (if yes, mention the name of the area……………………… |
| 2.11 | When did you diagnose COVID negative?  (Please write the date) | Days/month/year |
| 2.12 | What kinds of treatment you have received during COVID-19 status? | 1= Medicine  2= Ventilation  3= Oxygen supplementation |
| 2.13 | Did you have to admit in ICU for COVID-19? | 1= Yes  2= No |
| 2.14 | Do you receive any Physiotherapy intervention after recovering from COVID-19 | 1= Yes  2= No  If yes, mention the name  3= Chest Physiotherapy  4= Other Physiotherapy interventions |

**4.Self-administered Comorbidity Questionnaire.**

(If you have any of these following comorbid diseases, please put √ on it. You may have multiple diseases; you can put √ on multiple points.)

|  | Do you have the problem? | | Do you receive treatment for it? | | Does it limit your activities? | |
| --- | --- | --- | --- | --- | --- | --- |
| **Problem** | No(0) | Yes(1) | No(0) | Yes(1) | No(0) | Yes(1) |
| Heart diseases | N | Y | N | Y | N | Y |
| High blood pressure | N | Y | N | Y | N | Y |
| Lung disease | N | Y | N | Y | N | Y |
| Diabetes | N | Y | N | Y | N | Y |
| Ulcer and stomach disease | N | Y | N | Y | N | Y |
| Kidney disease | N | Y | N | Y | N | Y |
| Liver disease | N | Y | N | Y | N | Y |
| Anemia or other blood diseases | N | Y | N | Y | N | Y |
| Cancer | N | Y | N | Y | N | Y |
| Depression | N | Y | N | Y | N | Y |
| Osteoarthritis, Degenerative arthritis | N | Y | N | Y | N | Y |
| Back pain | N | Y | N | Y | N | Y |
| Rheumatoid arthritis | N | Y | N | Y | N | Y |
| Other medical conditions.  (specify) | N | Y | N | Y | N | Y |
|  | N | Y | N | Y | N | Y |
|  | N | Y | N | Y | N | Y |
| **Total:** |  | | | | | |

**5. Brief-COPE (Brief-COPE) Questionnaire**

The following questions ask how you have sought to cope with a hardship in your life. Read the statements and indicate how much you have been using each coping style.

|  | I haven't been doing this at all | A little bit | A medium amount | I’ve been doing this a lot |
| --- | --- | --- | --- | --- |
| 2.1 I've been turning to work or other activities to take my mind off things. | 1 | 2 | 3 | 4 |
| 2.2 I've been concentrating my efforts on doing something about the situation I'm in. | 1 | 2 | 3 | 4 |
| 2.3 I've been saying to myself "this isn't real". | 1 | 2 | 3 | 4 |
| 2.4 I've been using alcohol or other drugs to make myself feel better | 1 | 2 | 3 | 4 |
| 2.5 I've been getting emotional support from others. | 1 | 2 | 3 | 4 |
| 2.6 I've been giving up trying to deal with it. | 1 | 2 | 3 | 4 |
| 2.7 I've been taking action to try to make the situation better. | 1 | 2 | 3 | 4 |
| 2.8 I've been refusing to believe that it has happened. | 1 | 2 | 3 | 4 |
| 2.9 I've been saying things to let my unpleasant feelings escape. | 1 | 2 | 3 | 4 |
| 2.10 I’ve been getting help and advice from other people. | 1 | 2 | 3 | 4 |
| 2.11 I've been using alcohol or other drugs to help me get through it. | 1 | 2 | 3 | 4 |
| 2.12 I've been trying to see it in a different light, to make it seem more positive. | 1 | 2 | 3 | 4 |
| 2.13 I’ve been criticizing myself. | 1 | 2 | 3 | 4 |
| 2.14 I've been trying to come up with a strategy about what to do. | 1 | 2 | 3 | 4 |
| 2.15 I've been getting comfort and understanding from someone. | 1 | 2 | 3 | 4 |
| 2.16 I've been giving up the attempt to cope. | 1 | 2 | 3 | 4 |
| 2.17 I've been looking for something good in what is happening. | 1 | 2 | 3 | 4 |
| 2.18 I've been making jokes about it. | 1 | 2 | 3 | 4 |
| 2.19 I've been doing something to think about it less, such as going to movies, watching TV, reading, daydreaming, sleeping, or shopping. | 1 | 2 | 3 | 4 |
| 2.20I've been accepting the reality of the fact that it has happened. | 1 | 2 | 3 | 4 |
| 2.21I've been expressing my negative feelings. | 1 | 2 | 3 | 4 |
| 2.22I've been trying to find comfort in my religion or spiritual beliefs. | 1 | 2 | 3 | 4 |
| 2.23I’ve been trying to get advice or help from other people about what | 1 | 2 | 3 | 4 |
| 2.24I've been learning to live with it. | 1 | 2 | 3 | 4 |
| 2.25 I've been thinking hard about what steps to take. | 1 | 2 | 3 | 4 |
| 2.26I’ve been blaming myself for things that happened | 1 | 2 | 3 | 4 |
| 2.27 I've been praying or meditating | 1 | 2 | 3 | 4 |
| 2.28 I've been making fun of the situation. | 1 | 2 | 3 | 4 |
| **Total** |  | | | |

**6.WHO-QoL BREF** (Put circle on your answer)

Please read each question, assess your feelings about your life in the last two weeks and circle the number on the scale for each question that gives the best answer for you.

|  |  | Very poor | Poor | Average (Neither poor nor good) | Good | Very good |
| --- | --- | --- | --- | --- | --- | --- |
| 1 | How would you rate your quality of life? | 1 | 2 | 3 | 4 | 5 |

|  |  | Very dissatisfied (not satisfied) | A little dissatisfied | Satisfied | Fairly satisfied | Very satisfied |
| --- | --- | --- | --- | --- | --- | --- |
| 2 | How satisfied are you with your health? | 1 | 2 | 3 | 4 | 5 |

The following questions ask about how much you have experienced certain things in the last two weeks.

|  |  | Very much/a lot | Quite a lot | Not at all/Average | A little | Very little |
| --- | --- | --- | --- | --- | --- | --- |
| 3 | To what extent do you feel that physical pain prevents you from doing what you need to do? | 1 | 2 | 3 | 4 | 5 |
| 4 | How much do you need any medical treatment to function in your daily life? | 1 | 2 | 3 | 4 | 5 |
| 5 | How much do you enjoy life? | 1 | 2 | 3 | 4 | 5 |
| 6 | To what extent do you feel your life to be meaningful? | 1 | 2 | 3 | 4 | 5 |

|  |  | Very little | A little | Average | Quite a lot | Very easily/extremely safe/healthy environment |
| --- | --- | --- | --- | --- | --- | --- |
| 7 | How well are you able to concentrate? | 1 | 2 | 3 | 4 | 5 |
| 8 | How safe do you feel in your daily life? | 1 | 2 | 3 | 4 | 5 |
| 9 | How healthy is your physical environment? | 1 | 2 | 3 | 4 | 5 |

The following questions ask about how completely you experience or were able to do certain things in the last two weeks.

|  |  | Very little | A little | Average | Quite a lot | Very much/a lot |
| --- | --- | --- | --- | --- | --- | --- |
| 10 | Do you have enough energy for everyday life? | 1 | 2 | 3 | 4 | 5 |
| 11 | Are you able to accept your bodily appearance? | 1 | 2 | 3 | 4 | 5 |
| 12 | Have you enough money to meet your needs? | 1 | 2 | 3 | 4 | 5 |
| 13 | How available to you is the information that you need in your day-to-day life? | 1 | 2 | 3 | 4 | 5 |
| 14 | To what extent do you have the opportunity for leisure activities? | 1 | 2 | 3 | 4 | 5 |

|  |  | Very little | A little | Not at all/Average | Quite a lot | Very easily/much |
| --- | --- | --- | --- | --- | --- | --- |
| 15 | How well are you able to get around? | 1 | 2 | 3 | 4 | 5 |

The following questions ask you to say how good or satisfied you have felt about various aspects of your life over the last two weeks.

|  |  | Very poor | Poor | Average | Good | Very good |
| --- | --- | --- | --- | --- | --- | --- |
| 16 | How satisfied are you with your sleep? | 1 | 2 | 3 | 4 | 5 |
| 17 | How satisfied are you with your ability to perform your daily living activities? | 1 | 2 | 3 | 4 | 5 |
| 18 | How satisfied are you with your capacity for work? | 1 | 2 | 3 | 4 | 5 |
| 19 | How satisfied are you with yourself? | 1 | 2 | 3 | 4 | 5 |
| 20 | How satisfied are you with your personal relationships? | 1 | 2 | 3 | 4 | 5 |
| 21 | How satisfied are you with your sex life? | 1 | 2 | 3 | 4 | 5 |
| 22 | How satisfied are you with the support you get from your friends? | 1 | 2 | 3 | 4 | 5 |
| 23 | How satisfied are you with the conditions of your living place? | 1 | 2 | 3 | 4 | 5 |
| 24 | How satisfied are you with your access to health services? | 1 | 2 | 3 | 4 | 5 |
| 25 | How satisfied are you with your transport? | 1 | 2 | 3 | 4 | 5 |

The following question refers to how often you have felt or experienced certain things in the last two weeks.

|  |  | Very little | A little | Not at all/average | Quite a lot | Very frequently/a lot |
| --- | --- | --- | --- | --- | --- | --- |
| 26 | How often do you have negative feelings such as blue mood, despair, anxiety, depression? | 1 | 2 | 3 | 4 | 5 |
